# A possible link between sinusitis and lower airway hypersensitivity: the role of Staphylococcal enterotoxin B

**DOI:** 10.1186/1476-7961-4-7

**Published:** 2006-05-07

**Authors:** Tao Liu, Bin-Quan Wang, Ping-Chang Yang

**Affiliations:** 1 Institute of Allergy and Department of Otolaryngology, the First Hospital, Shanxi Medical University, Taiyuan, China; 2 Department of Pathology and Molecular Medicine, McMaster University, Hamilton, ON, Canada

## Abstract

**Background and aims:**

The prevalence of asthma has been keeping arising with unknown etiology. The cumulative evidence indicates that chronic rhinosinusitis (CRS) closely relates to asthma, but the detailed mechanisms remain unclear. The present study aimed to take insight into the role of Staphylococcus enterotoxin B (SEB) in a possible association between CRS and asthma.

**Methods:**

38 patients with both CRS and asthma underwent functional endoscopic sinus surgery. Serum specific IgE and cytokines, clinical symptoms of CRS and asthma were evaluated before and after the surgery. Peripheral blood mononuclear cells (PBMCs) were separated from the patients and cultured. Th2 response of the cultured PBMCs in the presence or absence of specific antigens and SEB was evaluated.

**Results:**

Besides the improvement of CRS symptoms, amelioration of asthma was also observed in the patients with both CRS and asthma after the sinus surgery. The preoperatively elevated Th2 cytokines, IL-4 and IL-5, normalized postoperatively. Th2 response was generated with separated PBMCs in the presence of specific antigens. SEB was required for maintaining Th2 response in these separated PBMCs.

**Conclusion:**

The present results indicate that a possible link exists between CRS and lower airway hypersensitivity. Sinusitis derived SEB may play a role in sustaining Th2 responses in the low airway hypersensitivity related to sinusitis.

## Background

Asthma is a chronic respiratory disease characterized by episodes or attacks of inflammation and narrowing of small airways in response to exposure to environmental stimuli [1]. Asthma attacks can vary from mild to life-threatening. Asthma has been a significant health problem in the world, with an ever-increasing morbidity and mortality, and economic impact through both direct and indirect costs over the last 20 years [2]. Although the research in asthma has advanced rapidly in recent years, the etiology of asthma remains unclear.

The cummulative evidence suggests that CD4+ T-helper (Th)2 lymphocytes play a crucial role in the pathogenesis of asthma. Th-2 inflammatory response has been documented to predominate over Th1 response in the patients with asthma. Significant correlation has been identified between high levels of interleukin (IL)-4 and IL-5 and clinical severity of asthma [3,4]. The role of Th2 cytokines is of importance because IL-4 and IL-13 can promote IgE production [5]. Antigen specific IgE is a key molecule in mediating antigen-related asthma [6]. However, how the established Th1/Th2 balance breaks down, how the skewed Th2 polarization last for a long time in asthma patients remains to be understood.

A close relationship between chronic rhinosinusitis (CRS) and asthma has been noticed for decades [7,8]. In clinical practice, we have noticed that many patients who suffer from asthma or allergic rhinitis report the pre-existed CRS. The clinical data have demonstrated that surgical and non-surgical CRS management can improve clinical symptoms of CRS and asthma [9-11]. These data implicate that substance derived from the sinus infection may play a role in the pathogenesis of asthma with unknown mechanism.

Although the causes of CRS are complicated, it is accepted that bacterial infection is one of the causes in most circumstances. *Staphylococcus aureus *(*S. aureus*) is a common pathogen contributing to both acute and chronic rhinosinusitis [12]. It produces exotoxins such as Staphylococcal enterotoxin B (SEB), SEA and toxic shock syndrome toxin-1 (TSST-1), which possess potent immune activity to affect immune system. Reports demonstrate high levels of SEB and SEA in nasal secretions of patients with CRS and asthma [13]. The exotoxins have the potential in regulating airway immune reactions during CRS by contaminating airway mucosa through secretions from the sinus ostia [14]. SEB is capable of increasing airway epithelial barrier permeability, facilitating macromolecular protein antigen crossing of the epithelium to contact immune cells in subepithelial region [15].

SEB induces vigorous activation, proliferation, and cytokine production by T cells that express the specific TCR variable beta (Vβ) chains. Some investigators associate SEB with inducing the Th1 pattern inflammation [16-18], however, the accumulative evidence indicates that SEB is also involved in the pathogenesis of allergic diseases [19-22]. We and others have shown that the simultaneous exposure to SEB and food antigens enhances susceptibility to allergic reactions [14,23]. SEB triggers immune cells to release Th2 cytokines IL-4, IL-5 and IL-13 [14,24-26] and enhances antigen-specific immune responses [19,27]. Comparing atopy patients with healthy subjects, in the culture supernatants of PBMC with SEB stimulation, atopy patients produced more Th2 cytokines, such as IL-4 and IL-5, and less Th1 cytokines, such as interferon (IFN)-γ [28].

Our previous study [23] and others [29] show that the CRS can be an intrinsic source of SEB in the body. The rhinosinuses may be frequently contaminated and colonized by the *S. aureus *[12]. CRS derived SEB can be discharged with secretions to the nasal cavity and then be compelled backward to be swallowed down the gastrointestinal tract or to contaminate the airway mucosa.

Therefore, we hypothesized that CRS derived SEB might be a factor in the induction and maintenance of low airway hypersensitivity. In the present study, with a group patient suffering from both CRS and allergic asthma, their CRS was treated with functional sinus endoscopic surgery (FESS), we aimed to observe: (i) amelioration of clinical symptom of asthma; (ii) regulation of Th2 response and (iii) the role of SEB in the maintenance of the skewed Th2 polarization in separated PBMCs.

## Methods

### Subjects

This study was carried out at the Institute of Allergy and the Department of Otolaryngology of Shanxi Medical University. The study was approved by the local Ethical Committee. A written consent was obtained from each subject. Patients with both CRS and asthma were considered to be enrolled into this study. CRS diagnosis: at least a one-year history of clinical symptoms including purulent nasal secretions, postnasal secretions and middle meatus purulent secretions. Computed tomography (CT) scans showed evidence of maxillary sinusitis or/and ethmoid sinusitis. No underlying immunodeficiencies, cystic fibrosis, bronchiectasis, chronic obstructive pulmonary disease, diabetes mellitus, neoplasia, or fungal rhinosinusitis. Thirty-eight patients with both CRS and chronic asthma (21 females and 17 males, ranging from 16 to 48 years with a mean age of 28.5 years) were enrolled to this study. A group of 25 patients with CRS alone (male = 14; age = 26.8 ± 7.8) were enrolled into this study served as controls. All patients required surgical treatment since they had tried different medical remedies and had not obtained satisfactory results. Routine physical examinations were performed on each patient upon admittance.

These 38 patients also suffered from perennial asthma. All of them clearly stated that asthma symptoms occurred after the occurrence of sinusitis. The diagnosis of asthma was based on the reported guidelines [30]. The history of asthmatic symptoms included episodic dyspnea and wheezing. These symptoms could be controlled by inhaling β2-adrenoreceptor agonists, or theophyline preparations. Lung function tests were performed for patients with asthma before and 2 months after sinus surgery. A group of 23 patients with asthma alone (male = 12; age = 31.3 ± 8.3) were enrolled into this study served as asthma controls. Subjects were nonsmokers and not have used any inhaled corticosteroids for at least 2 months, or long-acting β2-agonists for at least 48 hours before antigen screening.

Twenty healthy subjects were enrolled into this study served as healthy controls (male = 10; age = 20.8 ± 0.5).

### Skin prick test

One drop of extract of each airborne antigen (1 mg/ml, purchased from Beijing Xiehe Hospital, Beijing, China) was applied to the patients' forearm. The skin covered by the drop of antigen was pricked with 1 mm single-peak lancets. The positive control was performed using 10 mg/mL histamine dihydrochloride; the negative control using saline solution. The results were read after 15 min. The outline of each weal was documented using a piece of transparent paper to copy the wheal [31]. The skin wheal areas were determined with scanned digital files using software ImageJ. Skin prick tests were regarded positive when the area was larger than 7 mm^2^.

### Inhaled allergen challenge

In order to determine the airway response to specific antigens, we chose the antigens to which the subject had had the largest wheal area on skin prick testing. At baseline, subjects underwent an inhaled incremental allergen challenge. Concentrations of 200 to 30,000 SQU/ml antigen extract (Beijing Xiehe Hospital, Beijing, China) were prepared in 0.9% saline. Baseline forced expiratory volume (FEV)1 was determined after inhalation of 0.9% saline, and then start from the lowest dose, the subject inhaled incremental concentrations of allergen. All inhalations were delivered through a breath-driven dosimeter by inspiring slowly from functional residual capacity to total lung capacity over 1 second and then holding breath for 6 seconds while wearing a nose clip. Five breaths were inhaled for each concentration. Inhalation of increasing concentrations of allergen continued until an early asthmatic response (EAR) (a decrease of >20% of saline baseline value) was achieved. FEV1 was then measured at the following times after allergen inhalation: 20, 40 and 60 minutes and 3, 5 and 8 hours. The later asthmatic response (LAR) was defined as a decrease in FEV1 from saline baseline value of more than 15% between 3 and 7 hours after allergen challenge.

### Serology

Blood samples were collected from each subject at admittance and two months after the FESS. The sera were separated and stored at -70°C until use. Levels of IL-4, IL-5, INF-γ, total immunoglobulin (Ig)E and specific IgE were determined by Enzyme-Linked Immunosorbent Assay (ELISA) (Genetimes Tech Inc. Shanghai, China).

### Scores of clinical symptoms

The clinical symptoms of both CRS and asthma were scored on a four-point scale: 0 for no symptoms, 1 for mild, 2 for moderate and 3 for severe. Symptoms of sinusitis included stuffed nose, nasal purulent discharge and sinusitis-caused headache. Symptoms of asthma included frequency of attack, wheezing and shortness of breath. An overall score of the symptoms was determined by the patients and their physicians before and after surgery as well as the follow-up visits.

### Treatment of CRS

All patients in this group were treated with FESS. The procedures of this surgery were referred to Kennedy's report [32,33]. The patients were asked to follow up in the clinic 0.5, 1, 2, 6 and 12 months after the surgical procedure. Necessary treatments were done in the follow-up visits or upon re-admitted into the hospital if required.

### Evaluation of SEB in sinus (or nasal) wash fluids (SWF)

Every patient with CRS underwent maxillary sinus puncturing and irrigating with saline. The SWF was collected prior to other procedures. 5 ml saline was injected into the sinus cavity and recollected and stored at -70°C until use. Protein content in SWF was evaluated with UV spectrophotometer (Beckman DU-65) at 280 nm. Contents of SEB, SEA and TSST1 (toxic shock syndrome toxin 1) in SWF were evaluated with ELISA (Biochem 21, Inc. Shanghai, China). Nasal lavage fluids (5 ml saline) instead of sinus irrigation were collected from the healthy controls and asthma controls. None of the subjects had acute upper respiratory acute infections within the past month.

### Identification of S. aureus in the sinuses

Surgically removed sinus tissues were obtained from each patient (a nasal lateral wall swab was obtained from asthma alone patients and healthy controls instead) and were sent to the Microbiological Laboratory within 30 minutes. The procedures were referred to out previous report [23].

### Antigen specific CD4+ cell proliferation assay

#### PBMC preparation

PBMCs were isolated from heparinized blood samples by density gradient centrifugation over Ficoll-Paque (Biochem 21, Inc. Shanghai, China). Cells were washed twice and resuspended in complete RPMI 1640 medium (Biochem 21, Inc. Shanghai, China), which was supplemented with L-glutamine, 10% fatal calf serum, 200 U penicillin/mL, 200 μg streptomycin solution/mL.

#### Allergen-induced PBMC proliferation studies

The separated PBMCs were cultured in triplicate at 5 × 10^5 ^cells/well in 200 μL media in the presence of anti-CD3 (10 μg/ml) and anti-CD28 (2 μg/ml) for 3 days before the addition of allergen. 1 μg/ml the strongest responsive antigen in the skin prick test (a mixture of the 15 antigens was used for the groups of healthy and CRS) was added to the culture. The culture continued for another 4 days. The supernatants were collected at the end of culture. The cytokines of Th1 (IFN-γ) and Th2 (IL-4) in the supernatants were determined with ELISA using commercial reagent kits (Biochem 21, Inc. Shanghai, China) following manufacturer's instruction. Another batch of the cells was cultured in the presence of ionomycin (300 ng/ml) and brefeldin (10 μg/ml) for internal staining in addition to the culture conditions above. These cells were fixed with 1% paraformaldehyde for 1 h at room temperature, stained with fluorescein isothiocyanate (FITC) labeled anti-IL-4 antibody (for Th2 cells) and phycoerythrin (PE) labeled anti-IFN-γ antibody (for Th1) for 30 min on ice. The positively stained cells were counted with flow cytometry. 20,000 events were analysed for each sample. Negative control staining was omit primary antibodies or stain with isotype Igs.

#### Role of SEB in the maintenance of Th2 reactions

Another batch of the separated PBMCs from each subject was cultured respectively in the presence of specific antigens as described above. SEB (20 μg/ml) was added to the culture on day 9 that lasted to the end of the experiments. The supernatants were sampled on day 1 and every 2 days. IL-4 levels in the supernatants were measured with ELISA as described above.

### Statistics

Data is expressed as means ± SD. Statistical significance was determined using the *student t *tests or the χ^2^tests. Significant difference was accepted when p values were less than 0.05.

## Results

### Staphylococcus aureus and SEB are identified in the sinuses of CRS patients

**Figure 1:**
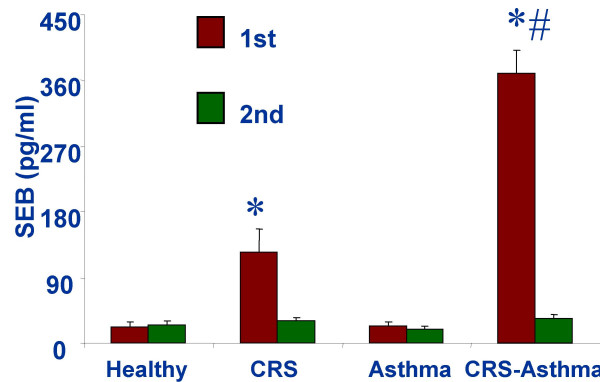
SEB was evaluated with ELISA in the sinus wash fluids. Bars represent levels of SEB as mean ± SD. *, p < 0.05, compared with healthy controls. #, p < 0.05, compared with the second time within the same group. 1^st^: samples are collected before the functional endoscope sinus surgery (FESS). 2^nd^: samples are collected 2 months after the FESS. Healthy: healthy controls. CRS: patients with chronic rhinosinusitis. Asthma: patients with asthma. CRS-asthma: patients with both CRS and asthma.

The surgical removed sinus tissues were subjected to *S. aureus *culture and the SWF samples were used to evaluate levels of SEB. 94.7% (36/38) CRS-asthma and 32% (8/25) CRS patients had *S. aureus *growth in the surgical removed tissues (χ^2 ^test: p < 0.05). CRS-asthma Patients showed much higher levels of SEB in the SWF that decreased significantly 2 months after FESS (Fig 1).

### Clinical symptoms of patients with CRS-asthma and CRS alone were improved after FESS

**Figure 2:**
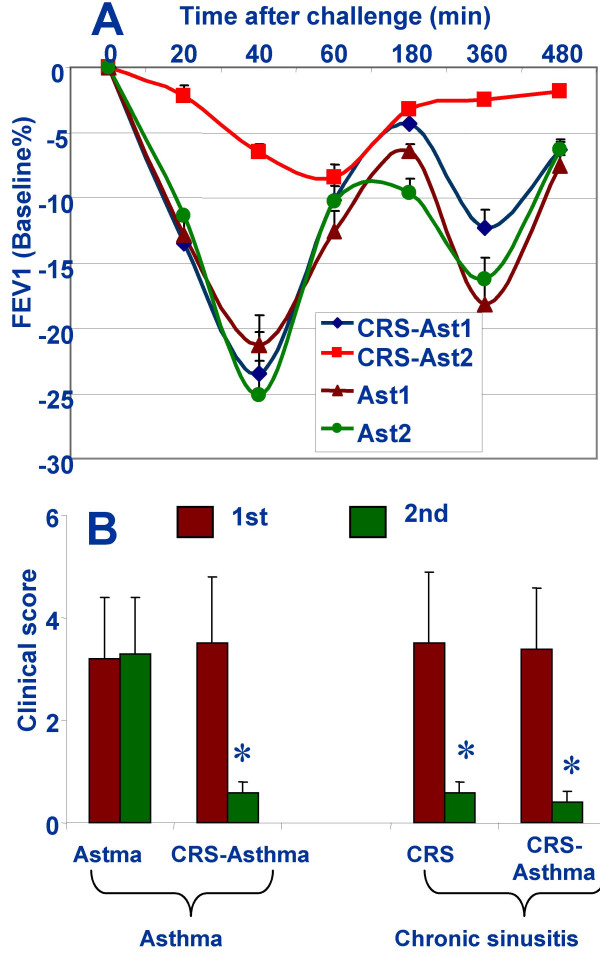
CRS and asthma clinical symptoms improved after the FESS. A, lung function after antigen challenge was assessed by recording FEV1 falling rate in the period of 0~8 h. B, overall clinical scores reported by patients. *, p < 0.05, compared with the 1^st ^scores within the same group.

Before the FESS, patients with CRS-asthma had a significantly lower baseline FEV1 value (2.58 ± 0.88, p < 0.01) compared with healthy controls (3.78 ± 0.56) and CRS controls (3.85 ± 0.48), but had no significant difference with asthma controls (2.61 ± 0.71). Two months after the FESS, FEV1 value fall-percentage in response to antigen challenge was also improved in CRS-asthma patients (improvement >12% could be considered as positive results), but not in patients with asthma alone (Fig 2A). The post-FESS clinical asthma scores decreased significantly (Fig 2B, the left penal). The clinical symptoms of CRS, such as nasal blockage and nasal purulent secretions were also assessed by patients. The FESS resulted in markedly attenuating the CRS clinical scores (Fig 2B, right side).

### Serum IgE and Th2 cytokine levels are repressed after FESS

**Figure 3:**
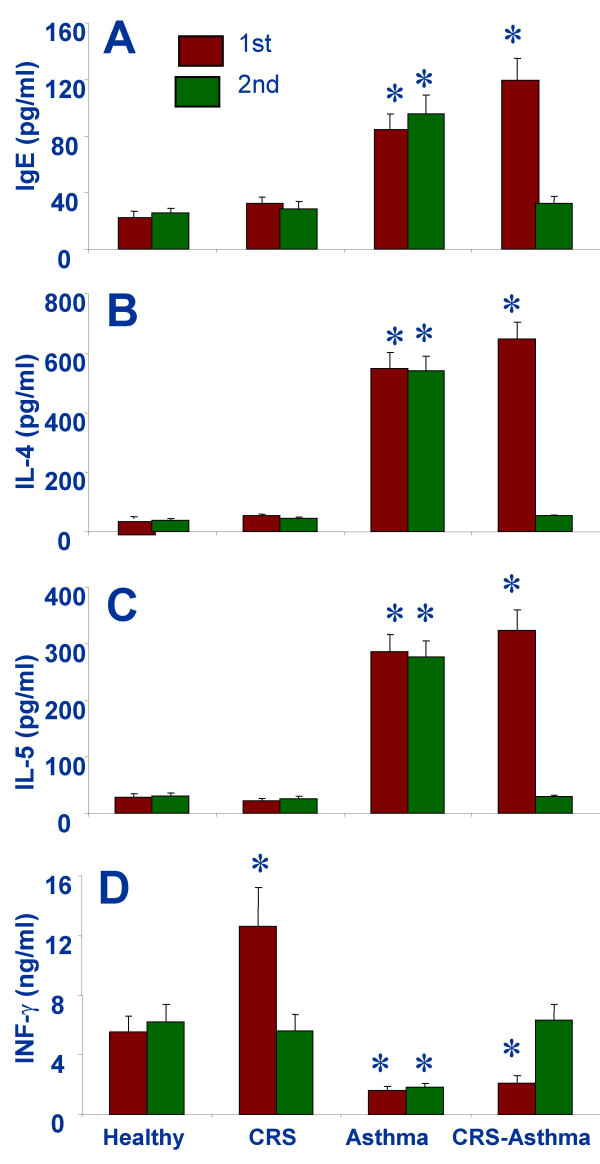
Serum specific IgE and Th1/Th2 cytokine levels were evaluated with ELISA. The serum samples were collected before (1^st^) and 2 months (2^nd^) after the FESS. *, p < 0.05, compared with the healthy controls.

As shown in Fig 3, before the FESS, serum cytokine assessment resulted in a Th2 skewed polarization profile and higher specific IgE levels in CRS-asthma and asthma alone patients. After the FESS, the levels of specific IgE, IL-4 and IL-5 were attenuated significantly in CRS-asthma patients, but not in asthma alone patients. There were no significant changes between the two assessments in the control groups.

### The skin prick test reactions decrease after the FESS

**Figure 4:**
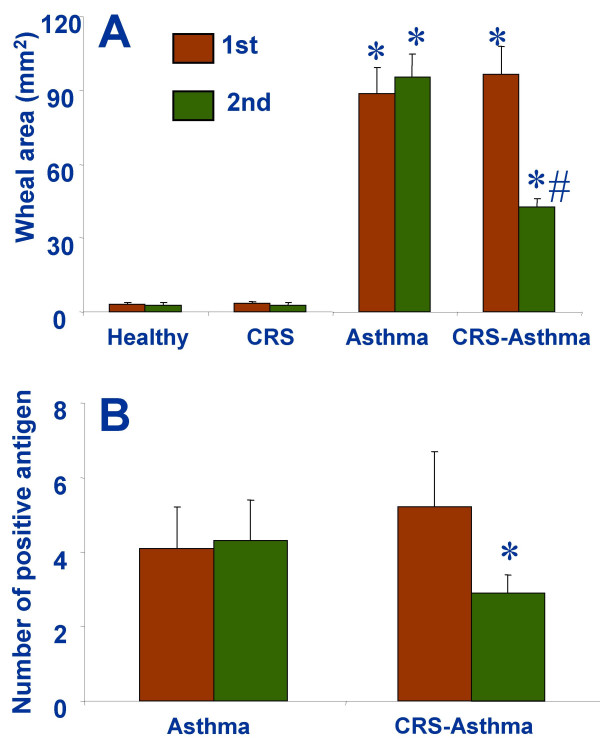
Antigen skin test was performed before (1^st^) and 2 months after the FESS (2^nd^). The skin wheal area was recorded by image processing technique to represent the antigen induced skin responses. *, p < 0.05, compared with the 1^st ^results (paired *student t-test*).

Each subject underwent two times of the skin prick test with a set of 15 common airborne antigens before and two months after the FESS. The results showed that the wheal area larger than 7 mm^2 ^was observed in the CRS-asthma patients in 1 to 6 antigens as depicted in Figure 4A that decreased significantly two months after the FESS. The number of the positive antigens in the CRS-asthma group was also reduced after the FESS (Fig 4B). The wheal area of the skin prick test of the patients with asthma alone was not significantly different from that of the CRS-asthma patients before FESS; the results did not show much difference between the two tests in asthma alone patients. The healthy control group did not respond to any tested antigens (the wheal area was less than 7 mm^2^), nor in patients with CRS alone. Saline was used as the diluent for all the antigens and was also used as the negative control. No special reactions were observed on the saline spots in all the subjectives.

### The antigen specific Th2 response is augmented by the exposure to SEB

**Figure 5:**
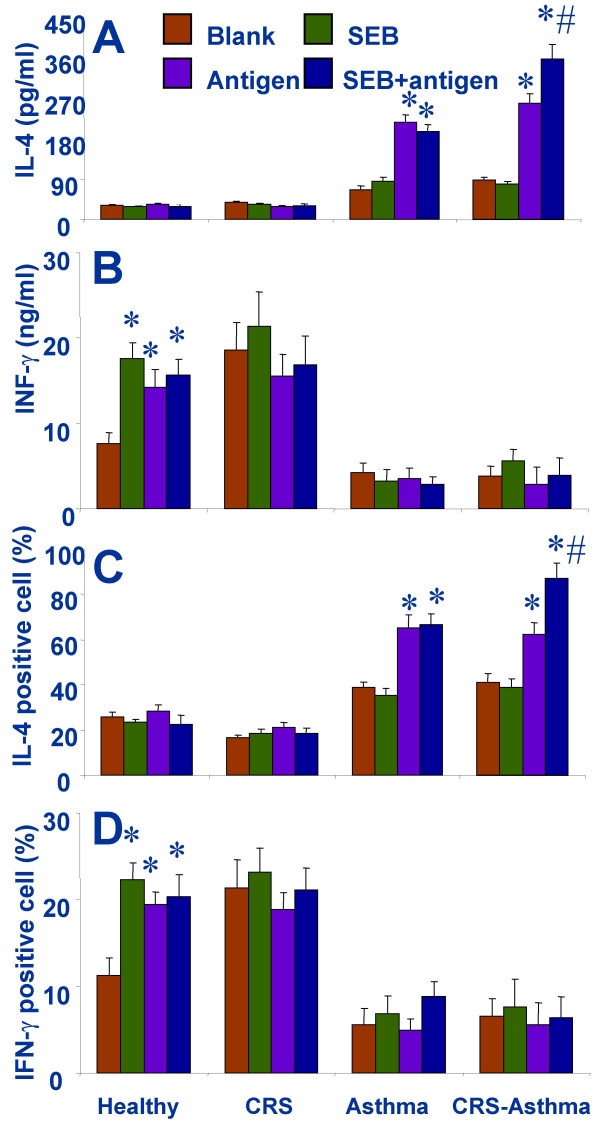
Th1/Th2 responses of the separated and cultured peripheral blood mononuclear cells (PBMCs) in response to specific antigen stimulation. IL-4 levels (A) represent Th2 response and IFN-γ levels (B) represent Th1 response. The IL-4 (C) and IFN-γ (D) positively stained cells were detected by immune staining and flow cytometry. Data are expressed as mean ± SD. *, p < 0.05, compared with no treatment cells.

As depicted in Fig 5, the results showed when the separated PBMCs from CRS-asthma patients were stimulated by specific antigens or SEB, the Th2 cytokine production increased significantly; a further increase was observed when the Th2 cells were stimulated with specific antigens and SEB concurrently. The PBMCs from asthma alone patients also showed an increase in Th2 profile response similar to that from CRS-asthma patients, but the Th2 profile did not further increase in the environment of SEB+antigen. The T cells from patients with CRS alone showed a Th1 profile with an increase in Th1 cytokine production, but that did not further increase in response to the stimulation of SEB or the mixed antigens or both. The separated PBMCs from the healthy controls were also stimulated with SEB or mixed antigens or both. The stimulation did not show any effect on their Th1/Th2 cytokine profiles.

### SEB plays a role in the maintenance of Th2 response

**Figure 6:**
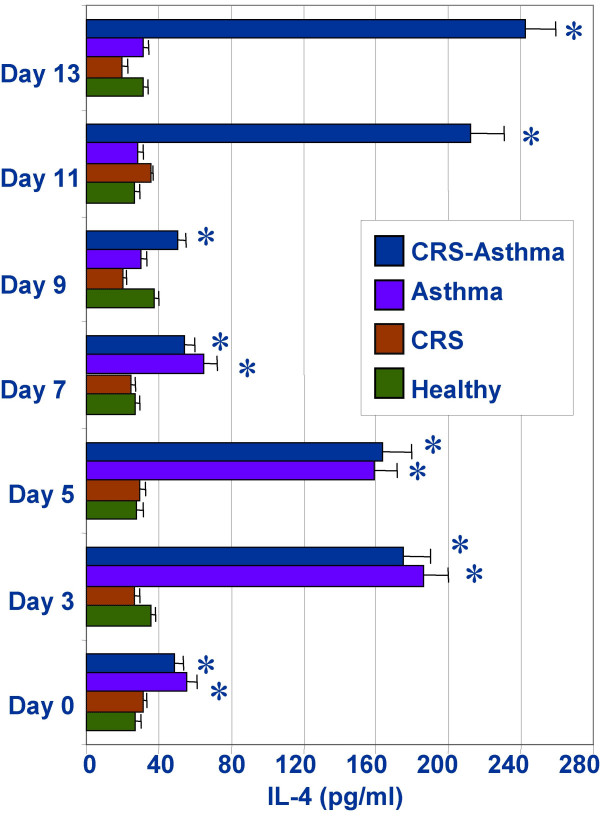
Time course of Th1/Th2 responses of the cultured PBMCs in response to stimulations of specific antigens and SEB. SEB was added to the culture on day 9. Data are expressed as mean ± SD. *, p < 0.05, compared with healthy controls.

The separated PBMCs were cultured with specific antigens in the presence or absence of SEB. In consistent with previous reports [34], we also observed an increase in IL-4 production in the samples from CRS-asthma and asthma alone patients on day 4. However, the IL-4 production declined rapidly afterwards. Considering SEB might be required in maintaining the immune activity of antigen specific Th2 cells, SEB was added to the culture. IL-4 production increased sharply in the cells from CRS-asthma patients, but not in the cells from asthma alone patients, or healthy controls and CRS alone patients (Fig 6).

## Discussion

The main findings of the present study include: (i) the remove of pathogen from the rhinosinuses improved clinical symptoms of patients with both CRS and asthma; (ii) SEB plays a role in the maintenance of the skewed Th2 polarization. The improvement of asthmatic symptoms and pulmonary function after FESS in patients with both CRS and asthma has been reported. The present data are compatible with previous reports. McFadden [35] observed a group patient with sinusitis and asthma undergoing sinus surgery and showed subjective improvement and increased respiratory function; 68 patients (85%) had significant improvement in their sinus symptoms and 67 (83%) had relief of their asthma. In a recent study, Tosca demonstrated an improvement in severity of asthma and respiratory functioning, together with reduced levels of inflammatory cells and a change from a Th2 cytokine profile to a Th1 profile [36]. Jankowski [37] reported on 30 patients and 91% showed asthma improvement. In the present study, the results approximately close to those reports as mentioned above, the follow-up results two month after the sinus surgery show that in addition to the clinical recovery of CRS, their asthma clinical symptoms had markedly improved as reported by the patients. The lung function test in response to antigen challenge is an objective test that demonstrates significantly improvement of the lung function of the patients with CRS-asthma after FESS (Fig 2). The results and the supporting literature suggest that rhinosinusitis might be associated with the pathogenesis of the low airway hypersensitivity, at least in a part of patients with CRS-asthma. Some investigators reported different results. Uri reported that the asthma symptoms were actually not improved although 88% patients felt better after the FESS [38].

The skin antigen tests show a significant decrease in the wheal area and the positive antigen number 2 months after the FESS. The results implicate a decrease in the amount of antigen specific IgE molecules binding on the surface of mast cells in the body. This reasoning is supported by the result of specific IgE evaluation that decreased significantly after the FESS. Another supportive datum is the FEV1 falling value was also improved in patients with CRS-asthma 2 months after the FESS, because the antigen specific IgE-bearing mast cells are the basis in the antigen induced low airway hypersensitivity. Similar results can be found in the reports of immunotherapy of asthma patients that shows markedly specific IgE repression as well as improvement of clinical symptoms of asthma after specific antigen immunotherapy [39].

We may ask a question: Why could FESS decrease the number of positive antigen in skin prick tests? As the in vitro study with PBMC culture shows, SEB is required in maintaining Th2 cell activity. Th2 cytokines IL-4 and IL-13 play a crucial role in IgE production. Theoretically, the more IgE production, the more IgE molecules bind on the surface of mast cells, thus the more intensive reaction of the mast cells can be in response to specific antigen challenge, the more inflammatory mediators to be released from the mast cells that cause more intensive reaction in antigen skin test. When the pathogen, *S. aureus*, has been removed from the sinuses, less SEB is produced. Therefore, the extent of antigen skin test reaction can be attenuated to the levels beyond the discriminative capability of the evaluation method.

The in vitro results show a skewed Th2 polarization profile in patients with CRS-asthma that is consistent with previous reports [40]. In allergic responses, B cells are driven to undergo an immunoglobulin isotype switch, shifting from IgM to IgE synthesis. This process involves the rearrangement of germline DNA in the immunoglobulin heavy-chain locus and is stimulated by cytokines (IL-4 and IL-13) and CD40 activation [41]. The decrease of serum IgE levels can be ascribed to the inhibition of Th2 cytokines as observed in this study. The function of IL-5 is associated with eosinophils development. The eosinophil is the critical effector cell type in inducing asthma clinical symptoms. Using IL-5 deficient animals can not develop the asthma models [42]. Therefore, the melioration of asthma clinical symptoms in CRS-asthma patients may be contributed by the reduction of IL-5 production, at least in part.

IL-4 and IL-5 are over produced by Th2 cells in allergic individuals. We also observed high levels of Th2 cytokines in the serum of the CRS-asthma patients. Because of IL-4 is also known to be produced by mast cells and basophils [43], the basophil is known increasing in asthmatic individuals [44], a possibility that the measured Th2 cytokines in the present study may also be produced by other cell types has not been ruled out. The present data emphasize the essential role of Th2 cell produced IL-4 and IL-5 in the induction of asthmatic clinical symptoms in patients with CRS-asthma. The separated PBMCs produced high levels of IL-4 when stimulated by specific antigens. These results strongly suggest that IL-4 is secreted by antigen-activated Th2 cells directly. The findings in this study also confirm that a large subset of asthmatics has reactivity to aeroallergens that is mediated by IL-4-secreting cells detectable in the circulation [45].

The antigen specific Th2 cells may be silence in out-season when the clinical symptoms of the season-type asthma also diminish. It is understandable that these cells lack of the specific stimulation from antigens. However, although the IL-4 production in the cultured PBMCs increases in the first 4 days, it declined gradually afterwards even though in the presence of specific antigens (Fig 6). The increase in IL-4 production in the antigen stimulated PBMCs is consistent with previous study [46]; the decline of IL-4 production in these antigen-activated PBMCs has gone to the opposite way against our expectation. Considering costimulators may be required in maintaining the activity of the antigen specific Th2 cells, we added SEB to these PBMCs since it is a T cell activator as reported before [47]. The IL-4 production increased again in the PBMCs from the patients with CRS-asthma, but not in patients with asthma alone, nor in other two control groups. Theses results have verified our postulation that SEB acts as a costimulator in maintaining the antigen specific Th2 cell activity at certain levels. The results support the advocation to remove any infectious foci especially in the airway may be a help in amelioration of asthma [9].

## Conclusion

In summary, we treated a group of patients with both CRS and Asthma with FESS. Significant amelioration of the clinical symptoms was obtained in both CRS and asthma. The separated PBMCs from patients with CRS and asthma show significant increase in production of Th2 cytokines in response to the specific antigen stimulation. SEB is required in maintaining the antigen specific Th2 cell activity in the in vitro study.

## Limitation of this study

This study has not proved that SEB is also required to maintaining the antigen specific Th2 cell activity in vivo. The symptoms of CRS and asthma were provided by the patients that might be bias in the results.

## Abbreviations

CRS: chronic rhinosinusitis; SEB: Staphylococcal enterotoxin B; FESS: functional endoscopic sinus surgery; PBMC: peripheral blood mononuclear cell; IL: interleukin.

## Competing interests

The author(s) declare that they have no competing interests.

## Authors' contributions

PY was involved in study design, a portion of FESS, histology, data analysis, some experiments and manuscript preparation; TL and BW were involved in CRS treatment, FESS and experiments.
